# Bismuth(III) Reagents in Steroid and Terpene Chemistry

**DOI:** 10.3390/molecules16042884

**Published:** 2011-04-04

**Authors:** Jorge A. R. Salvador, Samuel M. Silvestre, Rui M. A. Pinto

**Affiliations:** 1 Laboratório de Química Farmacêutica, Faculdade de Farmácia da Universidade de Coimbra, Pólo das Ciências da Saúde, Azinhaga de Santa Comba, 3000-548, Coimbra, Portugal; 2 Health Sciences Research Centre, Faculdade de Ciências da Saúde, Universidade da Beira Interior, Av. Infante D. Henrique, 6201-506 Covilhã, Portugal

**Keywords:** bismuth(III) salts, catalysis, steroids, terpenes, triterpenoids

## Abstract

Steroid and terpene chemistry still have a great impact on medicinal chemistry. Therefore, the development of new reactions or “greener” processes in this field is a contemporaneous issue. In this review, the use of bismuth(III) salts, as “ecofriendly” reagents/catalysts, on new chemical processes involving steroids and terpenes as substrates will be focused. Special attention will be given to some mechanistic considerations concerning selected reactions.

## 1. Introduction

Steroids [1] and terpenes [2] constitute a large and structurally diverse family of natural products and are considered important scaffolds for the synthesis of molecules of pharmaceutical interest. Different contributions ranging from natural product isolation and characterization to the synthesis of new compounds and their biological evaluation are reported every day. These investigations are justified by the well known biological properties of steroids and terpenes that make them useful molecules in pharmacy and medicine. 

The literature abounds with classical chemical reactions employed in steroid and terpene chemistry. Despite their wide applicability, these transformations often suffer from disadvantages such as handling of toxic, sensitive and/or expensive reagents, difficult work-up procedures, low yields and poor selectivities. Moreover, the lack of catalytic methods to perform a great number of reactions is evident.

The growing relevance of green and sustainable chemistry and the application of its guiding principles to the development of new reactions and chemical processes is changing the face of chemistry [3,4,5,6,7]. Several strategies have been developed for leading to more efficient, sustainable and environmentally friendly chemical processes and products. Among those strategies, catalysis and the design of new processes that avoid the use of toxic reagents have been the subject of intense research. In this context, bismuth(III) salts are suitable reagents for the design of “ecofriendly catalysts”, and a large number of methods involving the use of Bi(III) compounds in organic synthesis have been reported over the last decades [8,9,10,11,12,13,14,15,16,17,18,19,20,21,22]. Two review papers focusing on the recent advances of bismuth(III) salts usage in organic chemistry, with special emphasis on their application to the synthesis of compounds of pharmaceutical interest have quite recently been published [23,24].

In this review, the use of bismuth(III) salts as reagents and/or catalyst in reactions involving steroids and terpenes as substrates is presented. The review is organized by reaction type with emphasis on the following topics: oxidation reactions, formation and removal of common protecting groups, ring-opening of epoxides by nucleophiles, rearrangement reactions and miscellaneous reactions. Particular attention is given to works that highlight what could be called the hidden “Bi” behavior of bismuth. In fact, increasing evidence supports that the catalytic activity of bismuth(III) salts and its hydrates cannot be attributed to single Lewis acid activation of the substrates by bismuth cation, only. Indeed, the observed catalytic activities is thought to be attributable to a subtle balance between each bismuth species of Brønsted and/or Lewis acidic activation [25].

## 2. Oxidation Reactions

### 2.1. Oxidation of α-hydroxyketones by Bi_2_O_3_

**Scheme 1:**
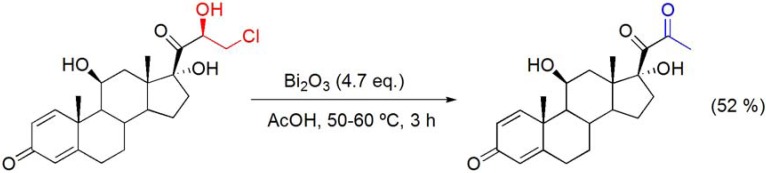
Bi_2_O_3_/AcOH oxidation of the 20,21-ketol group of a 21-chloromethylpregnane derivative.

The use of Bi_2_O_3_, in refluxing acetic acid was found to be an efficient and selective oxidant for the oxidation of α-hydroxyketones to the corresponding diones. According to Rigby, the true oxidant is presumably Bi(OAc)_3_, formed *in situ* under the reaction conditions, and is subsequently reduced to elemental bismuth (dark powder) [26,27]. This classical and versatile process has been extensively applied in the preparation of various steroids and terpenes, due, mainly, to its chemical selectivity. For example, this method has been useful in the synthesis of 11-hydroxy-12-keto- Δ^9(11)^-steroids with cholane [28,29], pregnane [30] and androstane [31] backbones. The oxidation of the α-hydroxyketone moiety present in ring A of several steroids with Bi_2_O_3_/AcOH was reported as an efficient way to obtain 2-hydroxy-3-keto-Δ^1^-steroids [32,33,34]. A different reactivity was observed in the Bi_2_O_3_/AcOHoxidation of the 20,21-ketol group of a 21-chloromethyl-pregnane derivative resulted in the formation of a dehydrohalogenated 20,21-diketo product, in 52% yield (**Scheme 1**) [35].

**Scheme 2:**
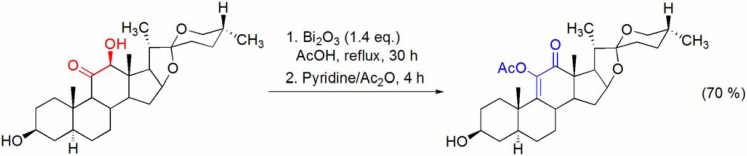
Bi_2_O_3_/AcOH oxidation of the 11-keto-12β-hydroxy moiety of a hecogenin derivative.

The Bi_2_O_3_/AcOH system has been applied to the preparation of an intermediate in the synthesis of cortisone starting from hecogenin, since under this reaction conditions the labile spirostan side chain remained intact (**Scheme 2**) [28,29].

**Scheme 3:**
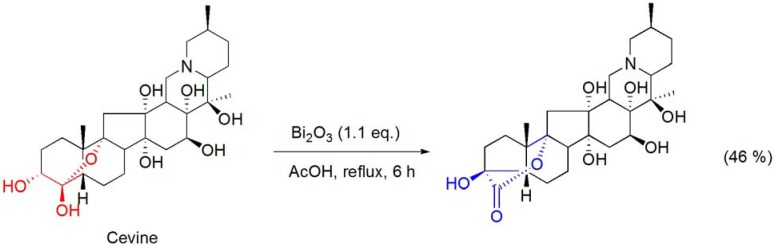
Bi_2_O_3_/AcOH oxidation of cevine.

The triterpene alkaloid cevine, bearing a hemiacetal function, was converted into a 5-membered ring A hydroxy-δ-lactone product by treatment with the Bi_2_O_3_/AcOH system (**Scheme 3**) [36]. Similarly, the conversion of the related triterpenes veracevine and cevagenine into similar hydroxy-δ-lactone derivatives was also reported by Kupchan and Lavie [36]. 

**Scheme 4:**
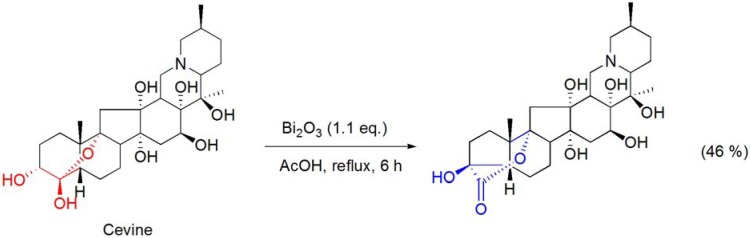
Bi_2_O_3_/AcOH oxidation of an intermediate for the synthesis of bruceantin.

Other examples of the application of this Bi_2_O_3_/AcOH system include the final step in the synthesis of diosphenolene [37], the preparation of α-diketo intermediate during the total synthesis of the racemate of aphidicolin [38] and the oxidation the α-hydroxyketone functionality present in ring A of highly oxygenated tetracyclic triterpenes, such as cucurbitacin D [39] and cucurbitacin B [40]. More recently, during the total synthesis of bruceantin, the Bi_2_O_3_/AcOH system was applied to the oxidation of an α-hydroxy ketone intermediate to afford the corresponding diosphenol, in 72% yield (**Scheme 4**) [41].

### 2.2. Allylic oxidation

**Scheme 5:**
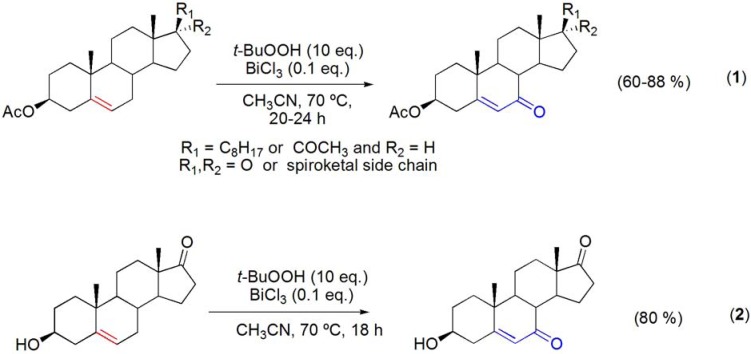
Bismuth (III) salt-catalyzed allylic oxidation of Δ^5^-steroids.

The allylic oxidation of Δ^5^-steroids [42,43] using several homogeneous or heterogeneous bismuth catalysts in combination with *t*-BuOOH has been recently reported [44,45]. BiCl_3_ was found to be the best catalyst and several Δ^5^-steroids were converted into the corresponding Δ^5^-7-oxosteroids in good to high yields (**Scheme 5**). The BiCl_3_/*t*-BuOOH system proved to be very selective for this reaction, since significant epoxidation of the double bond, secondary hydroxyl group oxidation (**Scheme 5, reaction 2**) or cleavage of the diosgenin side chain were not observed.

Moreover, a white insoluble solid was formed during the BiCl_3_-catayzed reactions. This solid was recovered by filtration and identified by X-ray diffraction (XRD) analysis as BiOCl. Thus, BiCl_3_ can be recovered at the end of the reaction and reused as BiOCl, which is also active under these reaction conditions, or reconverted again into BiCl_3_. 

**Scheme 6:**
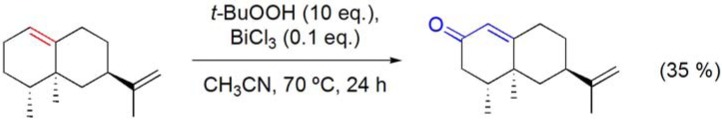
Bismuth (III) salt-catalyzed allylic oxidation of (+)-valencene.

Under similar reaction conditions, the allylic oxidation of (+)-valencene was reported and the corresponding α,β-unsaturated ketone, nootkatone, was obtained in 35% yield (**Scheme 6**) [44,45].

### 2.3. Oxidation of alcohols

**Scheme 7:**
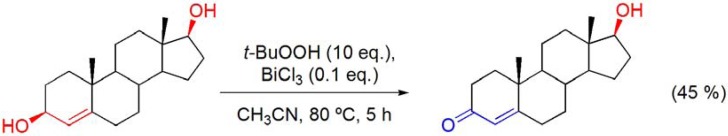
Selective allylic alcohol oxidation of androst-4-ene-3β,17β-diol by the BiCl_3_/*t*-BuOOH system.

Considering the excellent chemoselectivity observed in the allylic oxidation of dehydroepiandrosterone (**Scheme 5, reaction 2**), it was interesting to evaluate the selective allylic alcohol oxidation in the presence of a secondary saturated hydroxyl group using the BiCl_3_/*t*-BuOOH system. This study was performed using androst-4-ene-3β,17β-diol and it was observed that after complete consumption of this substrate, the important steroid hormone testosterone could be obtained, in 45% yield, after flash column chromatography (**Scheme 7**) [46].

**Scheme 8:**
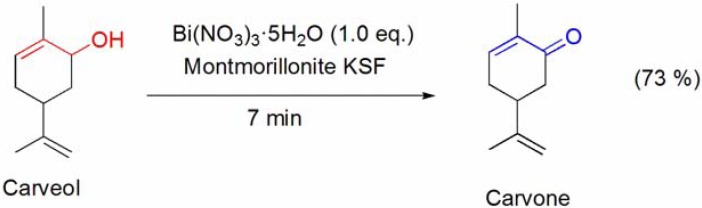
Oxidation of carveol with montmorillonite impregnated with Bi(NO_3_)_3_·5H_2_O.

In another study, the oxidation of the allylic alcohol moiety of carveol has been effected with montmorillonite impregnated with Bi(NO_3_)_3_·5H_2_O affording carvone, a naturally occurring monoterpene (**Scheme 8**) [47]. 

## 3. Formation of Common Protecting Groups

### 3.1. Acylation of alcohols

**Scheme 9:**
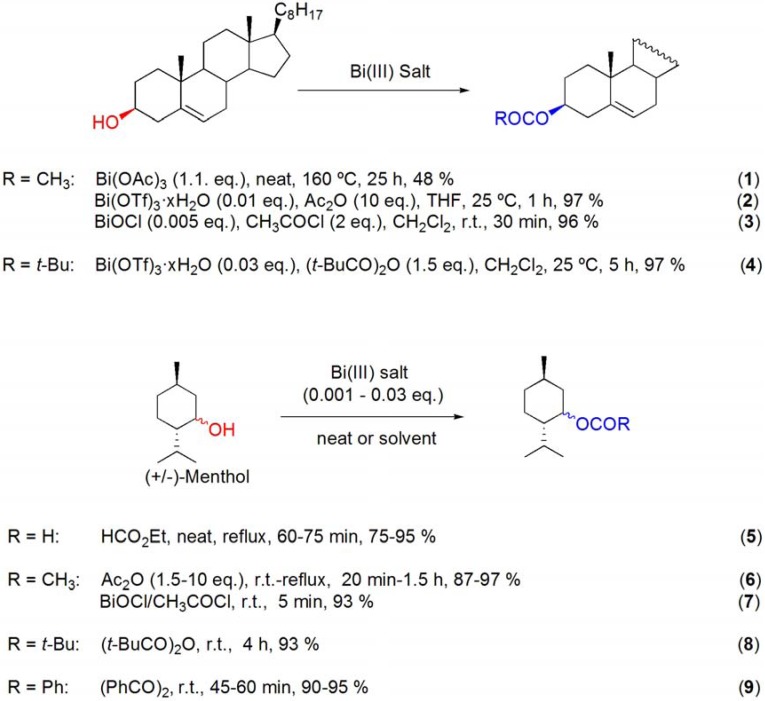
Different procedures for the acylation of cholesterol and menthol using bismuth(III) salts.

The conversion of cholesterol and cholesterol formate into the corresponding 3β-acetoxy derivative has been described by Reese and co-workers using stoichiometric amounts of Bi(OAc)_3_ (**Scheme 9, reaction 1**) [48].

More recently, acylation procedures using bismuth(III) salts as catalysts have been reported, and the acetylation of cholesterol was performed using Bi(OTf)_3_·xH_2_O in the presence of acetic anhydride (**Scheme 9, reaction 2**) [49,50]. Remarkably, all the three hydroxyl groups of cholic acid methyl ester were converted to the corresponding acetoxy groups in excellent yield, regardless of the sterically hindered 12α-hydroxy group [49,50]. 

The Ac_2_O/Bi(OTf)_3_·xH_2_O system has been applied to the acetylation of several monooxygenated monoterpenes, such as geraniol [49,50], borneol [49,50], linalool [49,50] and menthol (**Scheme 9, reaction 6**) [50,51,52,53]. BiCl_3_ and Bi(CF_3_COO)_3_ were also reported as efficient catalysts for the acetylation of menthol [52,53], however higher temperatures and/or reaction times were needed to achieve yields identical to those obtained with Bi(OTf)_3_·xH_2_O.

A very efficient process for the acetylation of cholesterol (**Scheme 9, reaction 3**) [54] and menthol (**Scheme 9, reaction 7**), among several other alcohols, using *in situ* generated BiCl_3_ from the procatalyst BiOCl and acetyl chloride was also described.

The pivaloylation of cholesterol (**Scheme 9, reaction 4**), borneol and menthol (**Scheme 9, reaction 8**) was accomplished by Orita and co-workers using Bi(OTf)_3_·xH_2_O (3 mol%) in the presence of either pivaloic anhydride or pivaloyl chloride [50].

**Scheme 10:**
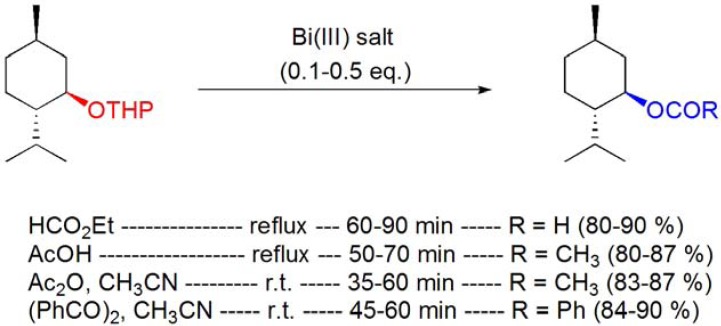
Bismuth(III) salt-catalyzed transformations of the THP ether of (–)-menthol to the corresponding formate, acetate and benzoate derivatives.

Menthol was quantitatively converted into the corresponding formate and benzoate derivatives after reaction with ethyl formate [53] and benzoic anhydride [52], respectively, in the presence of BiCl_3_, Bi(TFA)_3_ or Bi(OTf)_3_·xH_2_O (**Scheme 9, reactions 5 and 9**). Bismuth(III) salts catalyzed the direct conversion of the THP ether of menthol to the corresponding formate, acetate and benzoate derivatives by reaction with appropriate acylation reagents (**Scheme 10**). The reactions proceeded at reflux with ethyl formate or acetic acid [55], whereas the use of acetic or benzoic anhydrides, at room temperature, was enough to achieve high yields with short reaction times (**Scheme 10**) [56].

**Scheme 11:**
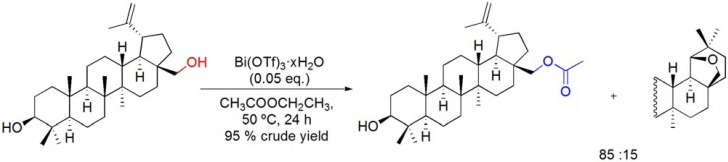
Transesterification of betulin with ethyl acetate catalyzed by Bi(OTf)_3_·xH_2_O.

An interesting transesterification of betulin with ethyl acetate has been recently reported, using Bi(OTf)_3_·xH_2_O as catalyst. Betulin-28-yl acetate was obtained as major reaction product along with allobetulin, resulting from the Bi(OTf)_3_·xH_2_O-catalyzed Wagner-Meerwein rearrangement (**Scheme 11**) [57].

### 3.2. Formation of ethers

**Scheme 12:**
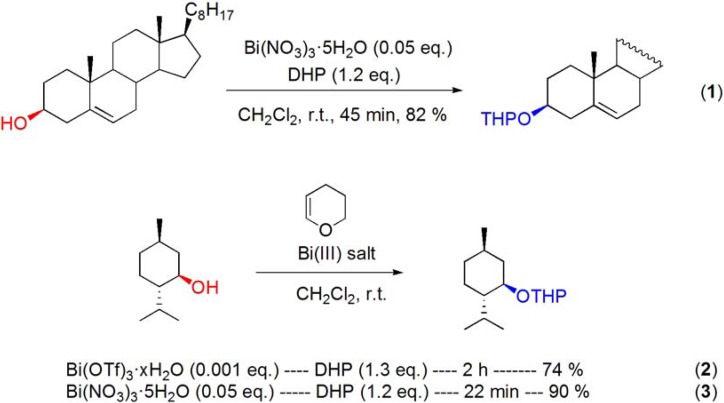
Bismuth(III) salt-catalyzed tetrahydropyranylation of cholesterol and menthol.

The tetrahydropyranylation of the secondary hydroxyl groups of cholesterol (**Scheme 12, reaction 1**), geraniol and (–)-menthol and (**Scheme 12, reaction 2**) and the tertiary alcohol of linalool has been accomplished by reaction with 3,4-dihydro-2*H*-pyran (DHP) in the presence of 0.1 mol% of Bi(OTf)_3_·xH_2_O [58]. More recently, Bi(NO_3_)_3_·5H_2_O was reported to be a more efficient catalyst, affording the tetrahydropyranyl (THP) ethers of menthol (**Scheme 12, reaction 3**) and geraniol in high yield, in shorter reaction times [59].

**Scheme 13:**
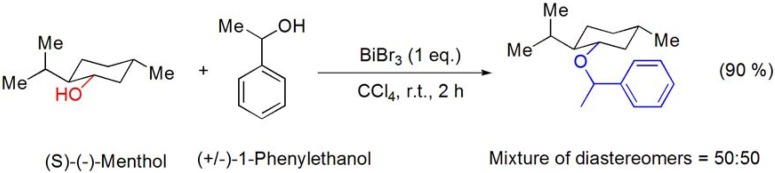
BiBr_3_-catalyzed benzylation of (*S*)-(–)-menthol.

Keramane and co-workers reported the benzylation of (S)-(–)-menthol with racemic 1-phenylethanol in the presence of catalytic amounts of BiBr_3_ [60]. The reaction was performed using the toxic CCl_4_ as solvent and the corresponding ether was obtained as an equimolar mixture of diastereomers in 90% yield (**Scheme 13**), and thus the reaction was shown to occur specifically with retention of configuration.

Very recently, a mild and efficient solvent-free method for the silylation of alcohols and phenols with hexamethyldisilazane (HDMS) and Bi(OTf)_3_·xH_2_O as catalyst was reported. This process was successfully applied to geraniol, an acid sensitive alcohol with two double bonds, and the corresponding trimethylsilyl ether (TMS) was prepared in 95% yield, after just 5 min. of reaction [61]. 

## 4. Removal of Common Protecting Groups

### 4.1. Deprotection of O,O-acetals

**Scheme 14:**
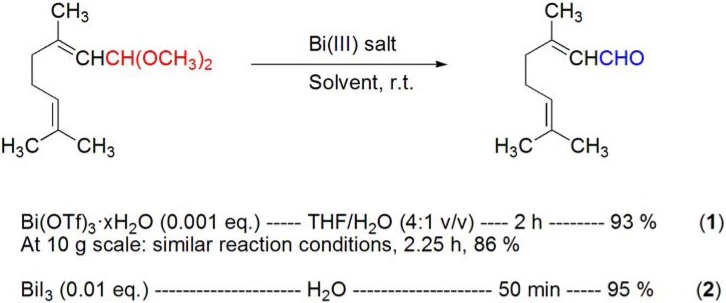
Bismuth(III) salt-catalyzed deprotection of citral dimethylacetal.

The deprotection of citral dimethylacetal has been performed in the presence of 0.1 mol % of Bi(OTf)_3_·xH_2_O using THF/H_2_O (4:1 v/v) as solvent (**Scheme 14, reaction 1**) [62]. More recently, the same reaction has also been reported with BiI_3_ (1 mol%) in H_2_O (**Scheme 14, reaction 2**) [63].

### 4.2. Deprotection of ether groups

**Scheme 15:**
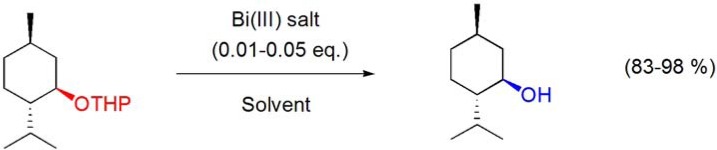
Bismuth(III) salt-catalyzed depyranylation reaction of (–)-menthol THP ether.

The high yielding depyranylation reactions of geraniol, menthol and linalool THP ether derivatives have been reported using 1 mol% Bi(OTf)_3_·xH_2_O in DMF-MeOH (9:1 v/v) [58] or BiCl_3_ (3 mol%), Bi(TFA)_3_ (5 mol%) and Bi(OTf)_3_·xH_2_O (1 mol%) in MeOH (**Scheme 15**) [64].

**Scheme 16:**
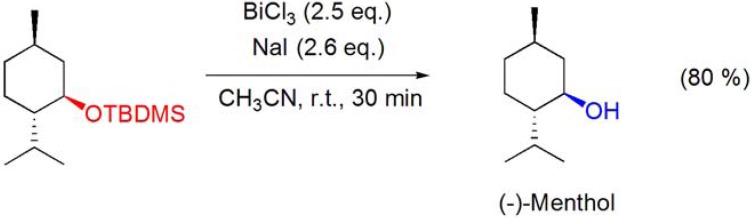
Deprotection of the *tert*-butyldimethylsilyl ether of (–)-menthol by the BiCl_3_/NaI system.

The deprotection of the *tert*-butyldimethylsilyl ether of (–)-menthol was accomplished using the BiCl_3_/NaI system in acetonitrile, at room temperature, in 80% yield, without loss of the original configuration (**Scheme 16**) [65].

### 4.3. Deprotection of oximes to carbonyl compounds

**Scheme 17:**
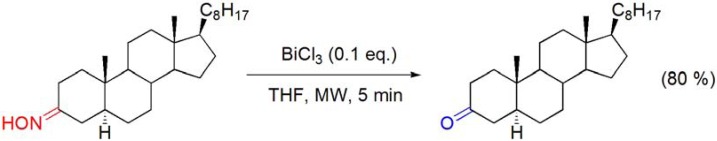
BiCl_3_-catalyzed conversion of 5α-cholestan-3-one oxime to the parent 3-keto-steroid.

A catalytic procedure using 10 mol% of BiCl_3_, in THF, under microwave irradiation, was reported for the regeneration of carbonyl groups from their oximes. The methodology was applied to 5α-cholestan-3-one oxime and the corresponding 3-keto-steroid was obtained in 80% yield (**Scheme 17**) [66].

## 5. Nucleophilic Ring-opening of Epoxides

### 5.1. Ritter reaction of epoxides

**Scheme 18:**
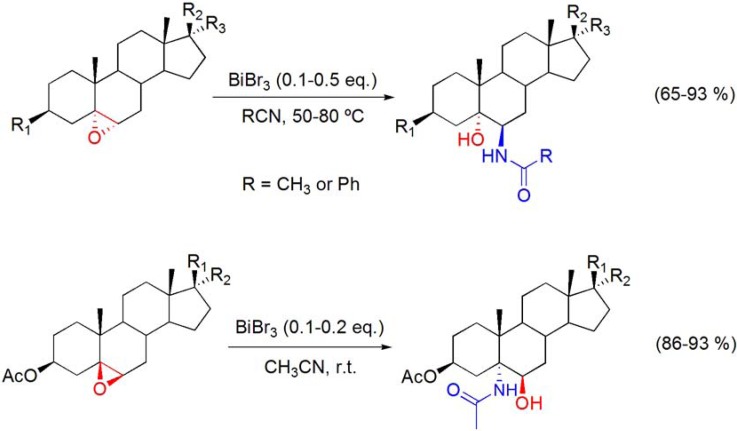
BiBr_3_-catalyzed Ritter reaction of epoxysteroids.

The Ritter reaction of 5α,6α- and 5β,6β-epoxysteroids with nitriles in the presence of bismuth(III) salts was reported to give the corresponding *vic*-acylamino-hydroxy products, in high yields (**Scheme 18**) [67,68,69]. This process was stereo- and regioselective as the result of the *trans*-diaxial ring-opening of the epoxysteroids. In the presence of several other functional groups, such as hydroxyl, ketone or ester, the reaction occurred selectively at the epoxide group. Interestingly, a quite recent study using DFT calculation showed that the higher affinity of Bi for Br can prevent side reactions [70] and makes BiBr_3_ a better Lewis acid catalyst, which is in accordance with the better results achieved with BiBr_3_ in this Ritter reaction, when compared with BiCl_3_. 

**Scheme 19:**
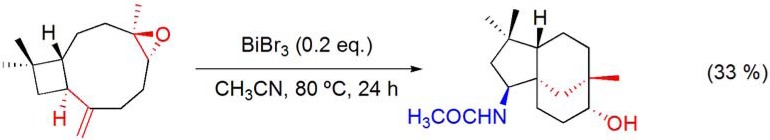
BiBr_3_-catalyzed Ritter reaction of (–)-caryophyllene oxide.

During the study of the bismuth(III)-promoted Ritter reaction of epoxides, the conversion of (–)-caryophyllene oxide into a clovan-9-ol derivative bearing a 2β-acetamide group at ring A was reported (**Scheme 19**) [67]. Despite the low yield (33%), this reaction is an alternative approach for the synthesis of clovane-type compounds with nitrogen atoms directly attached to C-2. 

### 5.2. Ring opening of epoxides with bismuth(III) salt

**Scheme 20:**
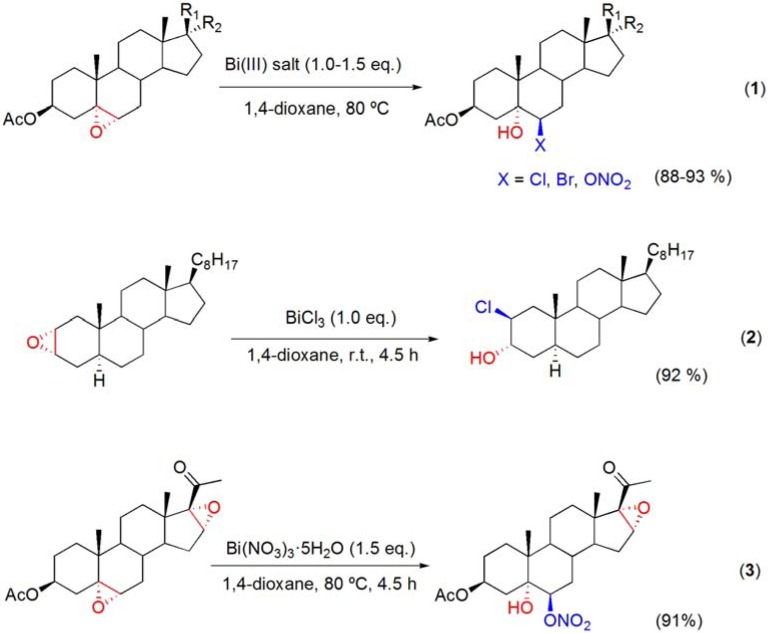
Ring opening of epoxysteroids with BiX_3_ (X = Cl, Br and ONO_2_).

The Ritter reaction of epoxysteroids was found to occur under catalytic and stoichiometric conditions with BiBr_3_ [67] However, when stoichiometric amounts of BiCl_3_ or Bi(NO_3_)_3_·5H_2_O were used to promote the Ritter reaction in acetonitrile, a competitive side product was detected, in both cases. These products were found to be the result of the epoxide ring-opening by BiCl_3_ and Bi(NO_3_)_3_·5H_2_O, respectively [71]. Thus, using stoichiometric amounts of bismuth(III) salts, such as BiCl_3_, BiBr_3_ or Bi(NO_3_)_3_·5H_2_O, and, simply, by changing the solvent to 1,4-dioxane, halohydrins and β-hydroxy-nitrates could be obtained in high yields by ring-opening of 5α,6α-, 5β,6β- and 2α,3α-epoxysteroids (**Scheme 20**) [71,72,73]. 

**Scheme 21:**
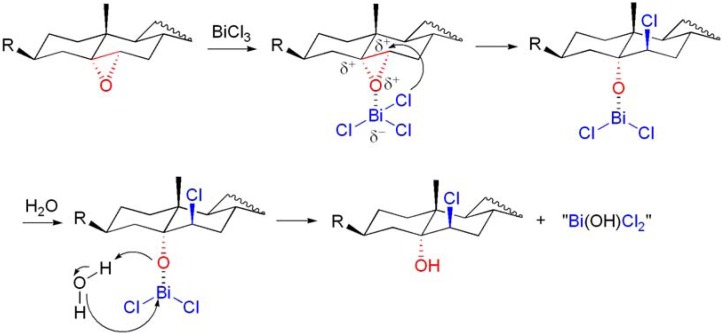
Possible mechanism for the ring opening of epoxysteroids with BiX_3_ (X = Cl, Br and ONO_2_).

The reactions were also stereo-, regio- and chemoselective. Notably, the ring opening of a 5α,6α;16α,17α-diepoxysteroid proved to be highly specific for the 5α,6α-epoxide group (**Scheme 20, reaction 3**) [71,73]. In order to understand the nature of the nucleophilic species, the reaction was performed in the presence of 2,6-di-*t*-butylpyridine (2,6-DTBP), a known proton scavenger which only binds to protons and is unable to coordinate to metal ions due to the bulky *t*-butyl groups [74]. Thus, in these reaction conditions, both BiCl_3_ and Bi(NO_3_)_3_·5H_2_O were able to afford the corresponding β-hydroxy substituted products, in a shorter reaction time than when the reaction was performed without 2,6-DTBP [71]. Taking into account the mechanism proposed by Keramane and co-workers for the chlorination of alcohols with BiCl_3_ [75] and a recent DFT work that studied the Lewis acidity of BiX_3_ (X = Cl, Br and I) towards alcohols [70], it has been suggested that the ring opening reaction is mediated by the Lewis acidity of bismuth towards the epoxide (**Scheme 21)**. As suggested by Keramane *et al*., a new bismuth species should be formed (“BiCl_2_OH”) [75]. In the ring opening of epoxides, one molecule of H_2_O, participates by furnishing a H^+^, present in the final hydroxyl group, whereas the OH^–^ will act as the third ligand of the newly formed bismuth species. Stabilization of Bi–O complex or the final “BiCl_2_OH” by 2,6-DTBP may help to explain the higher reaction rate observed in the reactions performed in the presence of this organic base.

### 5.3. Trans-diaxial hydroxylation of Δ^5^-steroids

**Scheme 22:**
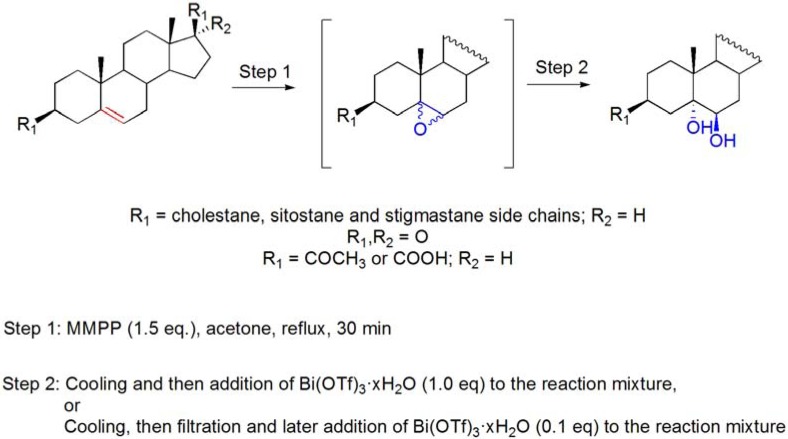
*Trans*-diaxial hydroxylation of Δ^5^-steroids by sequential combination of MMPP epoxidation and Bi(OTf)_3_·xH_2_O-catalyzed ring opening of epoxides.

Taking into account a recent report for the epoxidation of Δ^5^-steroids by the use of MMPP [76] and the previous knowledge of bismuth-catalyzed ring-opening reactions of epoxysteroids [67,71], Carvalho *et al*. described a fast and efficient high-yielding sequential approach for the preparation of 5α,6β-dihydroxysteroids using Δ^5^-steroids as raw materials [77]. This new synthetic protocol involves two steps: (i) formation of the epoxide from Δ^5^-steroids, using MMPP as oxidant; and (ii) *trans*-diaxial ring opening of the diastereomeric mixture of 5α,6α- and 65,6β-epoxides with Bi(OTf)_3_·xH_2_O in acetone. A catalytic ring-opening of the epoxide was achieved when a filtration was performed to remove the insoluble salts formed in the first step (**Scheme 22**) [77]. With this methodology, a library of 5α,6β-dihydroxy steroids has been easily obtained, and further research has provided important data regarding the cytotoxic profile of these compounds [78].

In this work, some considerations were done regarding the true catalytic species in the ring-opening step. Although the authors demonstrated the importance of Brønsted acid species, either derived from Bi(OTf)_3_·xH_2_O or from the oxidant, it has been suggested that Bi(OTf)_3_·xH_2_O also participates as Lewis acid in the catalytic cycle, since reactivity has been found, even when the reaction is performed in the presence of stoichiometric amounts of 2,6-DTPB [77].

## 6. Rearrangement Reactions

### 6.1. Ferrier rearrangement

The Ferrier rearrangement is a well known methodology for the preparation of alkyl and aryl 2,3-unsaturated-*O*-glycosides by reaction of glycals with alcohols catalyzed by Lewis acids. 

**Scheme 23:**
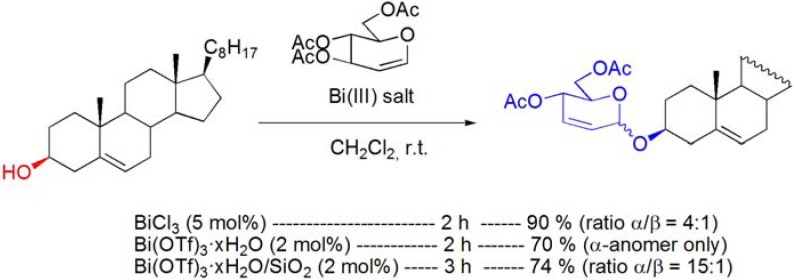
Bismuth(III) salt-catalyzed Ferrier rearrangement.

The reaction of cholesterol with 3,4,5-tri-*O*-acetyl-D-glucal in the presence of BiCl_3_ or Bi(OTf)_3_·xH_2_O afforded the corresponding 2,3-unsaturated-*O*-glycoside, in good yield [79,80]. The use of Bi(OTf)_3_·xH_2_O or Bi(OTf)_3_·xH_2_O/SiO_2_, proved to be more stereoselective, affording the α-anomer almost exclusively (**Scheme 23**) [80].

### 6.2. Epoxyolefin cyclizations

**Scheme 24:**
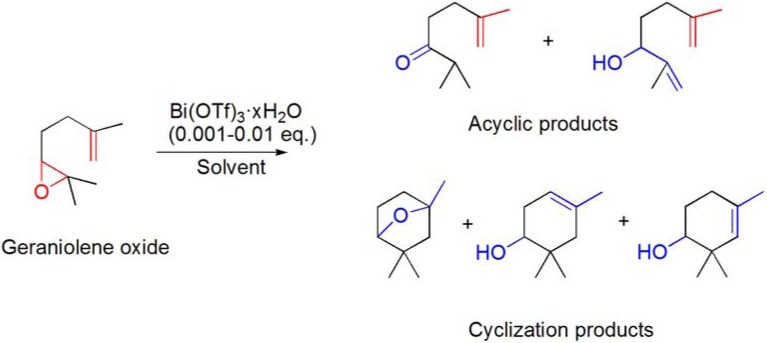
Bi(OTf)_3_·xH_2_O-catalyzed cyclization of geraniolene oxide.

Epoxyolefin cyclizations are an important area of research since the discovery that these reactions are involved in biosynthetic pathways of terpenes. The cyclization of geraniolene oxide has been investigated in the presence of several metal triflates, including Bi(OTf)_3_·xH_2_O. The reaction product ratio was found to be mainly influenced by the choice of the solvent and substrate concentration rather than by the choice of the metal triflate. Cyclization products were preferentially formed in CH_2_Cl_2_ and under high dilution conditions whereas acyclic compounds were mostly obtained in ethereal solvents (**Scheme 24**) [81].

Later, Smith and co-workers evaluated the use of stoichiometric amounts of BiCl_3_ and BiOClO_4_·xH_2_O in the Lewis acid mediated cyclization reactions of 6,7-epoxygeranyl pivalate ester also. Despite the fact that good activity was observed, poor selectivity for the desired bicyclic ether was found [82].

### 6.3. Westphalen and “Backbone” rearrangements

**Scheme 25:**
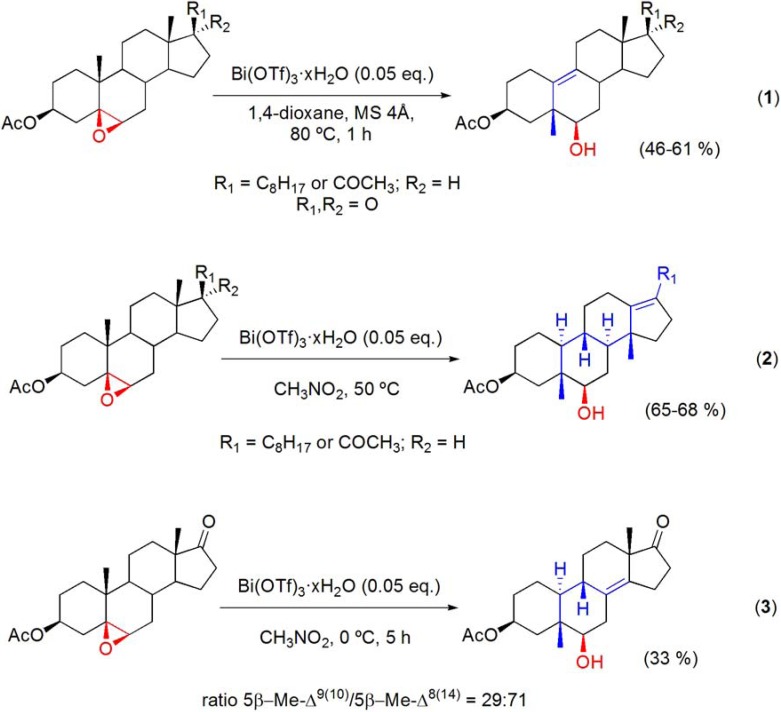
Bi(OTf)_3_·xH_2_O-catalyzed Westphalen and “backbone” rearrangements of 5β,6β-epoxysteroids.

Bismuth(III) salts were described as catalysts for the Westphalen and “backbone” rearrangements of 5β,6β-epoxysteroids [83,84]. The reactions were found to be particularly sensitive to changes on the solvent, temperature, stereochemistry of the starting epoxysteroids and their substituents at C-17. Thus, the Bi(OTf)_3_·xH_2_O-catalyzed reaction of 5β,6β-epoxysteroids from the cholestane, androstane and pregnane series, in 1,4-dioxane, at 80 ºC, afforded the corresponding 5β-methyl-Δ^9(10)^-19-norsteroids, in moderate yields (**Scheme 25, reaction 1**). On the other hand, using a solvent with high dielectric constant, such as CH_3_NO_2_, the rearrangement is extended to rings C and D of the steroid core and the 5β,14β-dimethyl-Δ^13(17)^-18,19-dinorsteroids from the cholestane and pregnane series were isolated in good yields (**Scheme 25, reaction 2**) [83,84].

**Scheme 26:**
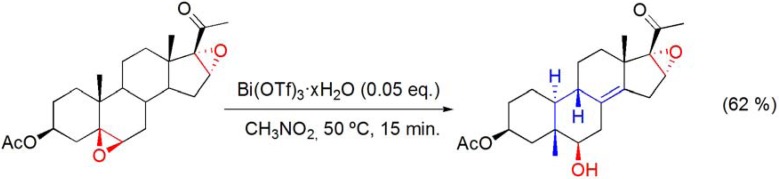
Bi(OTf)_3_·xH_2_O-catalyzed “backbone” rearrangement of 5β,6β;16α,17α-diepoxy-20-oxopregnan-3β-yl acetate.

With the androstane derivative, due to the lack of a 17α-H, a full “backbone” rearrangement was not expected and, in addition to the 5β-methyl-Δ^9(10)^-norsteroid derivative, the 5β-methyl-Δ^8(14)^-norsteroid was also formed, in Bi(OTf)_3_·xH_2_O/CH_3_NO_2_, and isolated in 33% yield (**Scheme 25, reaction 3**) [83]. An interesting feature of the “backbone” rearranged compounds is their abnormal A/B ring *trans*-fused (5β,10α) steroid structure that was confirmed by X-ray crystallography [83,84]. These rearrangements were found to occur specifically with 5β,6β-epoxides, and not with their corresponding 5α,6α-diastereomers. Data obtained from X-ray crystallography and quantum chemical calculation on the equilibrium geometry of the free molecule [84,85] revealed that important steric factors, such as the change in the torsion angle of the steroid nucleus, may contribute to explain the difference in reactivity observed between 5β,6β- and 5α,6α-epoxysteroids, which do not react under the same reaction conditions, although similar electronic effects are present in these diasteriomeric epoxides. Thus the 5β,6β-stereochemistry is essential for the development of 1,3-*syn*-diaxial interactions with the angular 10β-methyl group, that are relieved during the rearrangement. In another related study, it has been shown that the “backbone” rearrangement of the 5β,6β-epoxide group occurs preferencially to the Wagner-Meerwein rearrangement of the 16α,17α-epoxy-20-oxo moiety (**Scheme 26**) [86].

### 6.4. Wagner-Meerwein rearrangements

**Scheme 27:**
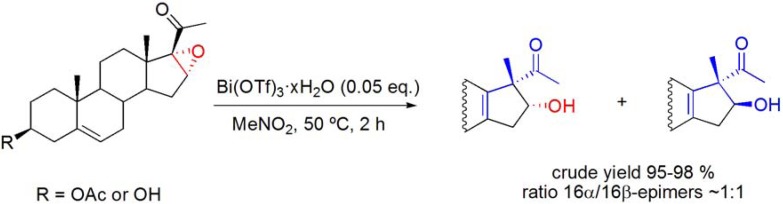
Bi(OTf)_3_·xH_2_O-catalyzed Wagner-Meerwein rearrangement of 16α,17α-epoxy-20-oxosteroids.

The application of Westphalen and “backbone” rearrangement reaction conditions [83] to 16α,17α-epoxy-20-oxosteroids has also been studied [86]. At room temperature, with 0.05 eq. of catalyst, low conversion of 16α,17α-epoxy-20-oxopregn-5-en-3β-yl acetate was observed. However, on increasing the temperature to 50 ºC, 16α- and 16β-hydroxy-17β-methyl-Δ^13^-18-nor pregnane derivatives were formed in near similar amounts from 16α,17α-epoxy-20-oxosteroids (**Scheme 27**) [86].

Several experiments, using HOTf or La(OTf)_3_ as catalysts instead of Bi(OTf)_3_·xH_2_O or with Bi(OTf)_3_·xH_2_O in presence of proton scavengers (2,6-DTPB and BiPh_3_), demonstrated that this rearrangement is mediated by Brønsted acid species generated *in situ* from the catalyst Bi(OTf)_3_·xH_2_O. It was also observed that the 16α-hydroxy-17β-methyl-Δ^13^-18-nor pregnane derivative undergoes epimerization under these reaction conditions.

**Scheme 28:**
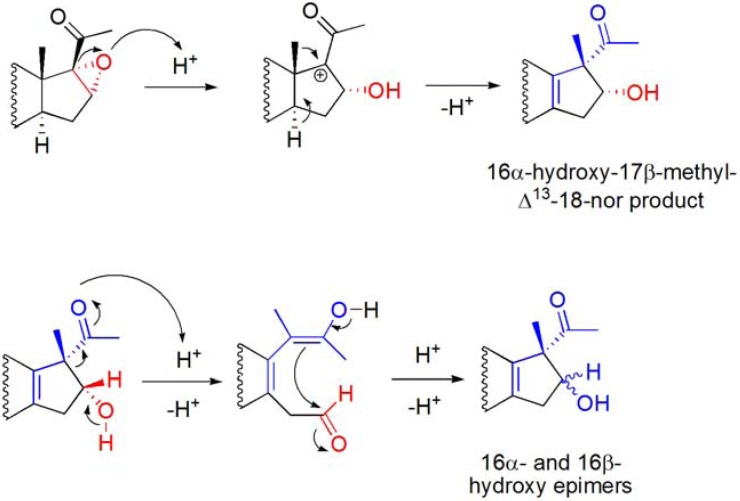
Possible mechanism for the formation of 16α- and 16β-hydroxy-17β-methyl-Δ^13^-18-norsteroids.

Thus, it was suggested that an *in situ* generated Brønsted acid species from Bi(OTf)_3_·xH_2_O catalyzes the ring opening of the 16α,17α-epoxide, creating a tertiary carbocation at C17 followed by stereoselective 1,2-migration of the C18-methyl group to the 17β-position. Due to the 18-CH_3_ → 17-CH_3_ shift, a carbocation centered at C13 is formed, and further 14α-H elimination originates the Δ^13^-double bond. 16β-Epimers can be formed due to an acid-catalyzed reverse-aldol equilibrium involving the 16-hydroxy-20-keto function of the rearranged steroid, under the reaction conditions employed, which is responsible for the epimerization at C16. (**Scheme 28**) [86].

**Scheme 29:**
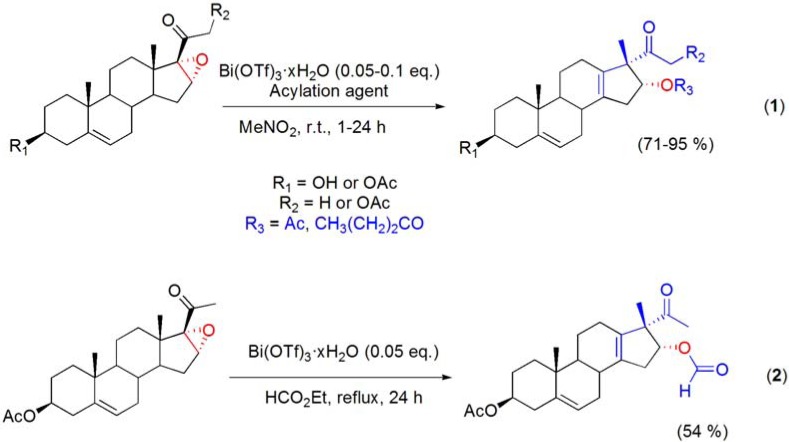
Bi(OTf)_3_·xH_2_O-catalyzed Wagner-Meerwein rearrangement in the presence of acylation reagents.

In order to increase the stereoselectivity of the rearrangement, the reactions were studied in the presence of Ac_2_O. In fact, it was observed that 16α-acetoxy rearranged derivatives were selectively prepared. To enlarge the scope of this Bi(OTf)_3_·xH_2_O-catalyzed rearrangement, other acylation agents were used, and several 17β-methyl-Δ^13^-18-norsteroids bearing different acyl groups at positions C3, C16 and C21 were selectively prepared (**Scheme 29**) [86].

**Scheme 30:**
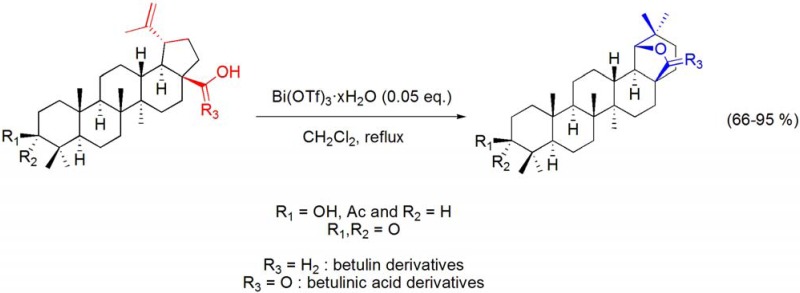
Bi(OTf)_3_·xH_2_O-catalyzed Wagner-Meerwein rearrangement of betulin and betulinic acid derivatives.

Wagner-Meerwein type rearrangements have also been widely reported in terpene chemistry [87]. One of such well-known transformations involves the acid catalyzed ring E rearrangement of betulin and important related triterpene derivatives, to 18α-oleanane compounds bearing a 19β,28-epoxide or 28,19β-lactone ring, and is commonly referred to “betulin-allobetulin rearrangement”. Following the previous knowledge that bismuth(III) salts are able to catalyze Wagner-Meerwein type rearrangements, their advantageous use as catalysts for the Wagner-Meerwein rearrangement of lupanes has recently been reported [57]. Thus, using CH_2_Cl_2_ as solvent and with Bi(OTf)_3_·xH_2_O catalysis (0.05 to 0.5 eq.), several lupane derivatives undergo a Wagner-Meerwein rearrangement with expansion of ring E and formation of an additional *O*-containing ring. By using this process [with 0.05 eq. of Bi(OTf)_3_·xH_2_O], several interesting 18α-oleananes could be efficiently obtained from lupanes by a single Wagner-Meerwein rearrangement (**Scheme 30**) [57,88].

**Scheme 31:**
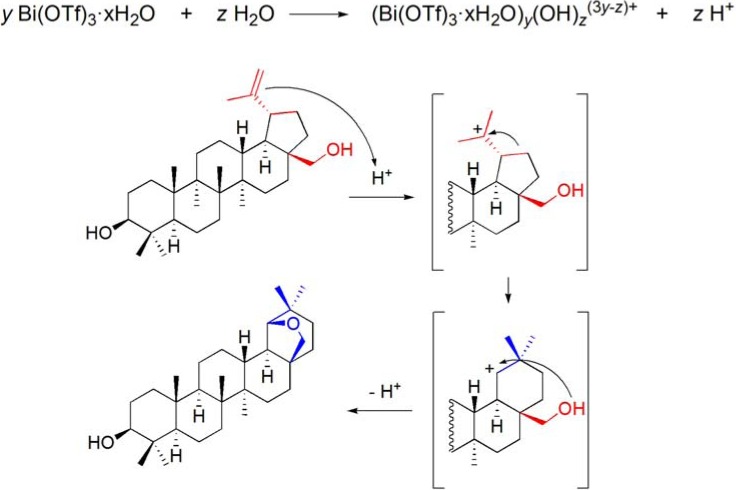
Possible mechanism for the Bi(OTf)_3_·xH_2_O-catalyzed Wagner-Meerwein rearrangement of betulin to allobetulin.

Some mechanistic studies were performed, in a similar fashion to what has been done during the study of the Wagner-Meerwein rearrangement of 16α,17α-epoxy-20-oxosteroids [86], and it was demonstrated that these reactions are catalyzed by Brønsted acid species generated *in situ* from the hydrolysis of Bi(OTf)_3_·xH_2_O. Therefore, the reaction mechanism can be postulated as follow. Under this reaction conditions, Bi(OTf)_3_·xH_2_O undergo a reversible hydrolysis leading to the formation of bismuth-containing cationic species [17] and thus the formation of an *in situ* Brønsted acid species. The hydrolysis of Bi(OTf)_3_·xH_2_O has been shown to lead to the quantitative formation of triflic acid, in water [89], however experimental data under this reaction conditions do not allowed to conclude it, unquivocally. Anyway, the Brønsted acid species formed *in situ* catalysis the formation of successive carbocations that are eventually trapped by intramolecular nuclophylic attach by the C28-hydroxyl group (**Scheme 31**) [57].

**Scheme 32:**
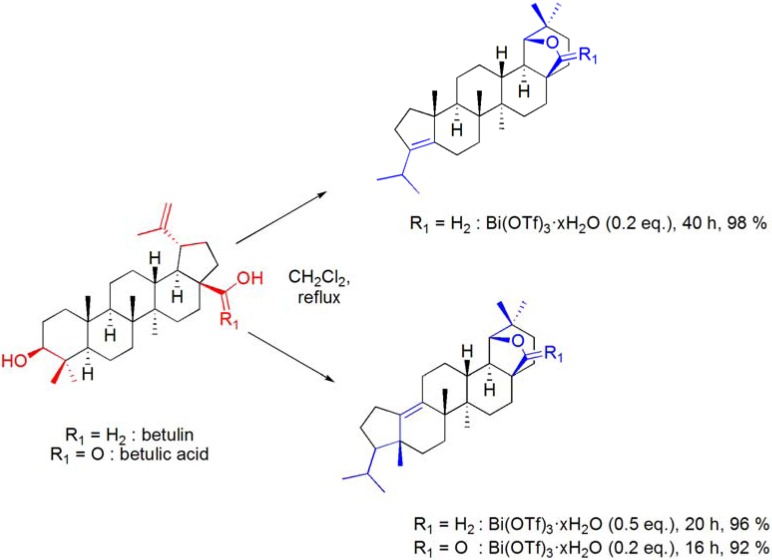
Bi(OTf)_3_·xH_2_O-promoted dehydration and double Wagner-Meerwein rearrangement of betulin (R_1_ = H_2_) and betulinic acid (R_1_ = O).

**Scheme 33:**
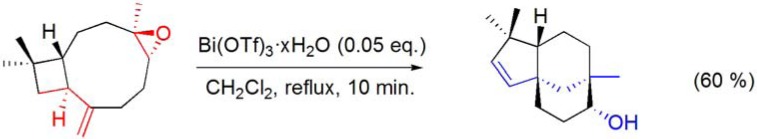
Bi(OTf)_3_·xH_2_O-catalyzed rearrangement of (–)-caryophyllene oxide.

Under more vigorous conditions (0.2-0.5 eq. of Bi(OTf)_3_·xH_2_O), the dehydration of the 3β-hydroxyl group occurred and the resulting compounds underwent a double Wagner-Meerwein rearrangement, originating A-neo-18α-oleanene compounds in high yields (**Scheme 32**). This process was also applied the sesquiterpene (–)-caryophyllene oxide, to afford clov-2-en-9α-ol by a “caryophyllene-clovane rearrangement” (**Scheme 33**).

### 6.5. Oxidative rearrangement of tertiary allylic alcohols

**Scheme 34:**
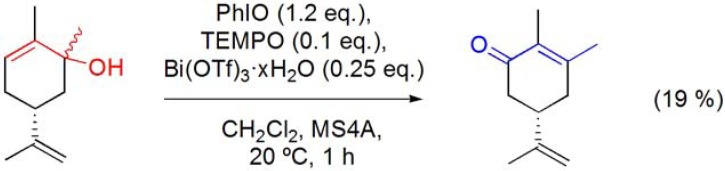
Bi(OTf)_3_·xH_2_O-catalyzed oxidative rearrangement of 2-methylcarveol.

Recently, a new method for the oxidative rearrangement of tertiary allylic alcohols to the corresponding transposed carbonyl derivatives using TEMPO in combination with PhIO and Bi(OTf)_3_·xH_2_O was described. However, when applied to the terpenic derivative 2-methylcarveol, this procedure only led to 19% yield of 3-methylcarvone **(Scheme 34)** [90,91].

## 7. Miscellaneous Reactions

### 7.1. Diels-Alder cycloaddition

**Scheme 35:**
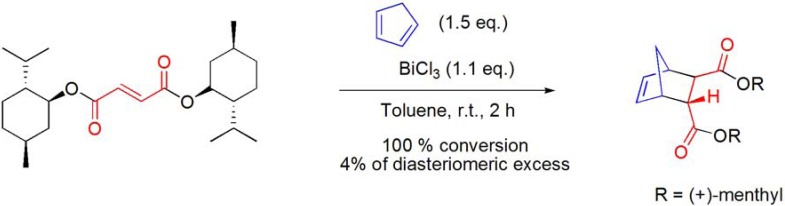
BiCl_3_-promoted Diels-Alder reaction of (+)-dimenthyl fumarate and cyclopentadiene.

(+)-Menthol was used as an inexpensive chiral auxiliary in the course of the development of a large-scale production process for the preparation of the enantiomerically pure (*2R,3R*)-bicyclo[2.2.1]hept-5-ene-2,3-dicarboxylic acid, an intermediate in the synthesis of the adenosine A1 antagonist BG9719 (CVT-124). In this context, he asymmetric Diels-Alder reaction of (+)-dimenthyl fumarate and cyclopentadiene has been extensively investigated in order to determine which conditions lead to the best yield and diastereomeric excess. Among the Lewis acids tested, BiCl_3_ promoted reaction rendered 100% conversion, but poor diastereomeric excess (**Scheme 35**) [92].

### 7.2. Ene reaction

**Scheme 36:**
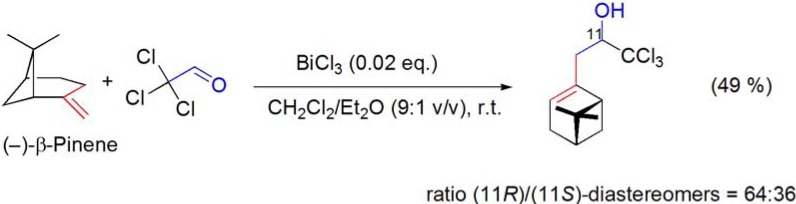
BiCl_3_-catalyzed ene reaction of (–)-β-pinene with chloral.

The BiCl_3_-catalyzed reaction of (–)-β-pinene with an equimolar amount of chloral in CH_2_Cl_2_/Et_2_O gave the corresponding H-ene adduct, in 49% yield [ratio (11*R*)/(11*S*)-diastereomers = 64:36] (**Scheme 36**) [93]. 

**Scheme 37:**
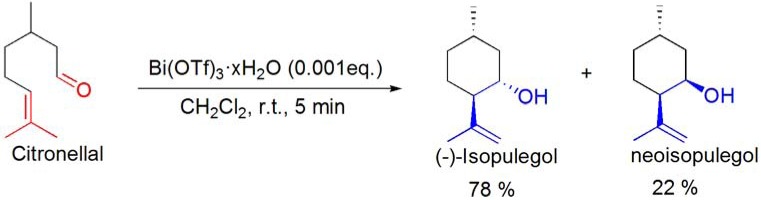
Bi(OTf)_3_·xH_2_O-catalyzed cyclization of citronellal.

A well known example of an intramolecular ene reaction is the cyclization of citronellal to (–)-isopulegol, an important intermediate in the industrial production of (–)-menthol (Takasago process). This reaction has been reported to be catalyzed by BiCl_3_ (2–5 mol%) [93] and Bi(OTf)_3_·xH_2_O (0.1 mol%) (**Scheme 37**) [94]. Along with the desired product, neoisopulegol was also formed in low amounts (<30%) in both processes.

### 7.3. Aromatic nitration reactions using Bi(NO_3_)_3_·5H_2_O

Montmorillonite impregnated with bismuth nitrate was found to be an efficient reagent for the nitration of estrone affording the 2-nitro and 4-nitro derivatives as a 1:1 mixture, in 94% yield [95,96]. 

**Scheme 38:**
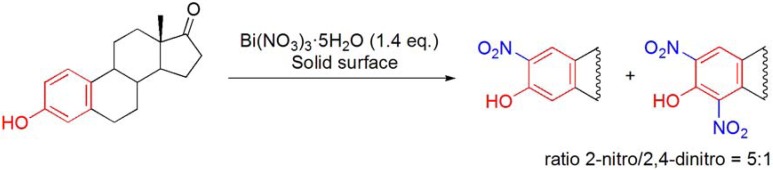
Nitration of estrone using Bi(NO_3_)_3_·5H_2_O.

More recently, the same group reported further developments of the initial methodology using various metal nitrates, including Bi(NO_3_)_3_·5H_2_O, and screened several solid supports and reaction conditions. The best results with Bi(NO_3_)_3_·5H_2_O were accomplished using Florisil or molecular sieves, in refluxing benzene, in a Dean Stark apparatus, or alumina, under dry conditions. With these reaction conditions, a 5:1 ratio of the 2-nitro and the 2,4-dinitro derivatives was obtained (**Scheme 38**) [97].

### 7.4. Lactonization of Δ^12^-oleananes

**Scheme 39:**
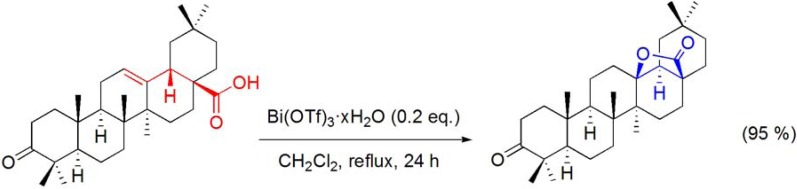
Bi(OTf)_3_·xH_2_O-promoted lactonization of oleanonic acid.

During the study of the Bi(OTf)_3_·xH_2_O-catalyzed Wagner-Meerwein rearrangement of terpenes, it was found that this reagent is able to promote the 28,13β-lactonization of oleanonic acid (**Scheme 39**) [57]. Interestingly, under bismuth catalysis, a carbocation is formed not only at C13 but also at C18, as shown by the observed inversion of the configuration of the C18-stereocenter, which has been confirmed by X-ray data [98].

### 7.5. Cleavage of the C17-side chain of corticosteroids

**Scheme 40:**
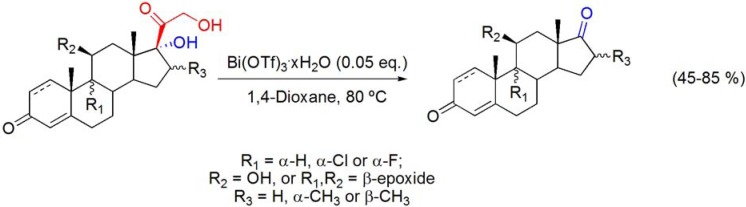
Bi(OTf)_3_·xH_2_O-catalyzed cleavage of the C17-dihydroxyacetone side chain of corticosteroids.

A quite recent report showed that Bi(OTf)_3_·xH_2_O is an efficient catalyst for the conversion of corticosteroids into highly functionalized 17-ketosteroids, by cleavage of the C17-dihydroxyacetone side chain [99]. This reaction was typically performed using large amounts of oxidative or basic reactants [100,101,102]. The use of other bismuth(III) salts and other solvents has been tested, however the best results have been obtained with 0.05 eq. of Bi(OTf)_3_·xH_2_O, in 1,4-dioxane, at 80 °C. Under this reaction conditions, non-steroidal by-products have been observed. Their presence probably arises from the acid-catalyzed polymerization of the α-hydroxy ketone moiety derived from the cleavage of the corticosteroid side chain. Thus, purification by flash chromatography was needed to obtain pure 17-ketosteroids. Using this method several highly functionalized 17-ketosteroids have been prepared (**Scheme 40**). 

The process proved to be very chemoselective, since functionalities of the starting corticosteroids, such as Δ^4^-3-keto, Δ^1,4^-3-keto, 11β-hydroxyl, and 9β,11β-epoxide, remained intact [99]. As an important drug class in the treatment of a variety of clinical situations [103], corticosteroids are readily available compounds. In fact, their industrial production reaches several tonnes per year [104], making this new process an useful source of substrates to obtain other steroids bearing a variety of chemical functions in rings A, B, C and D.

**Scheme 41:**
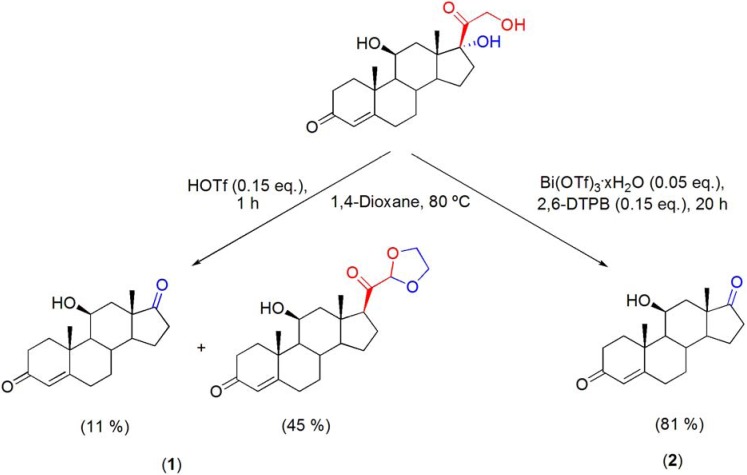
Cleavage of the C17-dihydroxyacetone side chain of corticosteroids under Brønsted acid and Lewis acid catalysis.

It is interesting to note that when the reaction was performed using HOTf as catalyst, only 11% of the 11β-hydroxy-17-ketosteroid was obtained along with 21-(1,3-dioxalan-2-yl)-11β-hydroxypregn-4-ene-3,20-dione, in 45% yield, as the major reaction product (**Scheme 41, reaction 1**). Moreover, using the Bi(OTf)_3_·xH_2_O/2,6-DTBP system, the desired product was obtained in good yield, after a longer reaction time (**Scheme 41, reaction 2**). Therefore, although Brønsted acid catalysis was observed, it seems the Bi(OTf)_3_·xH_2_O-catalyzed cleavage of the C17-dihydroxyacetone side chain of corticosteroids is mediated by Lewis acid catalysis [99]. In the absence of proton scavenger, Brønsted acid-assisted Lewis acid catalysis [25] is observed, as suggested by the higher reaction rate.

## 4. Conclusions

In the past five years, several new bismuth-based processes have been reported in the field of steroid and terpene chemistry, which added new “ecofriendly” tools for the synthesis of valorous molecules. 

Efforts have been made to tentatively identify the nature of the true catalytic species. Many literature reports attributed the reactivity to the Lewis acid nature of the bismuth(III) salts, however others explain the observed catalytic reaction as result of a Brønsted acid species formed *in situ* from bismuth(III) salt. In addition, Brønsted acid-assisted Lewis acid catalysis and Lewis acid-assisted Brønsted acid catalysis have also been found in selected transformations, involving bismuth(III) salts. This chemical behavior is responsible for many interesting reactivities of bismuth(III) salts towards steroids and terpenes. In fact, these catalysts were able to promote transformations that only have been earlier performed under extremely hard reaction conditions, such as strong mineral acids or powerful oxidant or basic reactants. 

Besides the great advances that have been obtained so far, more research in the development of new bismuth-based processes using steroid and terpenes of pharmaceutical interest is required. Moreover, incorporation of some of these procedures, in the design of large scale synthetic approaches for the preparation of selected steroid and terpenes, in a near future, will be expected.

## Supplementary Materials

Supplementary File 1
