# Supplementary material for: Bismuth(III) Reagents in Steroid and Terpene Chemistry
Source: Molecules. 2011 Apr 4;16(4):2884–913. doi: 10.3390/molecules16042884 (PMC6260628; doi:10.3390/molecules16042884)
Supplement: Supplementary File 1 [file molecules-16-02884-s001.pdf]

Molecules **2011**, *16*

OPEN ACCESS

molecules

ISSN 1420-3049

www.mdpi.com/journal/molecules

Correction

Salvador *et al*. Bismuth(III) Reagents in Steroid and Terpene Chemistry. *Molecules*, 2011, *16*, 2884-2913

Jorge A. R. Salvador [1,*], Samuel M. Silvestre [2] and Rui M. A. Pinto [1]

[1] Laboratório de Química Farmacêutica, Faculdade de Farmácia da Universidade de Coimbra, Pólo das Ciências da Saúde, Azinhaga de Santa Comba, 3000-548, Coimbra, Portugal

[2] Health Sciences Research Centre, Faculdade de Ciências da Saúde, Universidade da Beira Interior, Av. Infante D. Henrique, 6201-506 Covilhã, Portugal

* Author to whom correspondence should be addressed; E-Mail: salvador@ci.uc.pt; Tel.: +351 239488479; Fax: +351 239827126.

Received: 19 June 2011 / Published: 21 June 2011

The authors wish to make the following correction to this paper [1]: Scheme 4 is not correct. The corrected Scheme 4 is shown below:

Scheme 4. Bi_2O_3/AcOH oxidation of an intermediate for the synthesis of bruceantin.

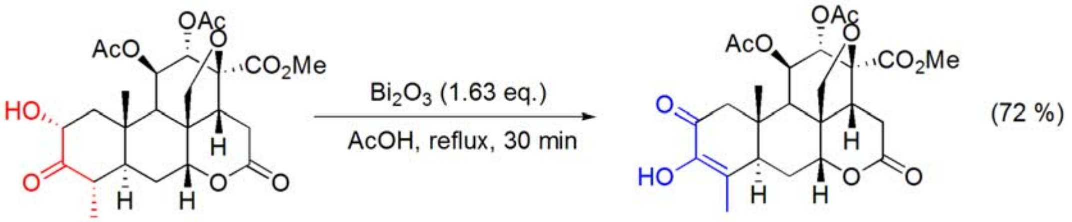

Reference

1. Salvador, J.A.R.; Silvestre, S.M.; Pinto, R.M.A. Bismuth(III) Reagents in Steroid and Terpene Chemistry. *Molecules* **2011**, *16*, 2884-2913.

© 2011 by the authors; licensee MDPI, Basel, Switzerland. This article is an open access article distributed under the terms and conditions of the Creative Commons Attribution license (http://creativecommons.org/licenses/by/3.0/).
